# What did we learn from the International Databases on Ambulatory and Home Blood Pressure in Relation to Cardiovascular Outcome?

**DOI:** 10.1038/s41440-023-01191-4

**Published:** 2023-02-03

**Authors:** Kei Asayama, Katarzyna Stolarz-Skrzypek, Wen-Yi Yang, Tine W. Hansen, Jana Brguljan-Hitij, Augustine N. Odili, Yan Li, Jan A. Staessen

**Affiliations:** 1 Department of Hygiene and Public Health, Teikyo University School of Medicine, Tokyo, Japan; 2 Research Unit Hypertension and Cardiovascular Epidemiology, KU Leuven Department of Cardiovascular Sciences, University of Leuven, Leuven, Belgium; 3 First Department of Cardiology, Interventional Electrocardiology and Hypertension, Jagiellonian University Medical College, Kraków, Poland; 4 Department of Cardiology, Shanghai General Hospital, Shanghai Jiao Tong University School of Medicine, Shanghai, China; 5 Steno Diabetes Center Copenhagen, the Capital Region of Denmark, Herlev, Denmark; 6 Division of Hypertension, Department of Internal Medicine, University Medical Center, Ljubljana, Slovenia; 7 Medical Faculty, University of Ljubljana, Ljubljana, Slovenia; 8 Department of Internal Medicine, Faculty of Clinical Sciences, College of Health Sciences, University of Abuja, Abuja, Nigeria; 9 Department of Cardiovascular Medicine, Shanghai Institute of Hypertension, Shanghai Key Laboratory of Hypertension, Ruijin Hospital, Shanghai Jiaotong University School of Medicine, Shanghai, China; 10 Non-Profit Research Association Alliance for the Promotion of Preventive Medicine, Leuven, Belgium; 11 Biomedical Science Group, Faculty of Medicine, University of Leuven, Leuven, Belgium

**Keywords:** Blood pressure, Cardiovascular complications, Hypertension, Individual Participant-Level Meta-analysis, Risk stratification

## Abstract

To assess in individual-person meta-analyses how out-of-office blood pressure (BP) contributes to risk stratification and the management of hypertension, an international consortium set up the International Databases on Ambulatory (IDACO) and Home (IDHOCO) Blood Pressure in Relation to Cardiovascular Outcome. This review summarizes key findings of recent IDACO/IDHOCO articles. Among various BP indexes derived from office and ambulatory BP recordings, the 24-h and nighttime BP level were the best predictors of adverse health outcomes. Second, using the 10-year cardiovascular risk associated with guideline-endorsed office BP thresholds as reference, corresponding thresholds were derived for home and ambulatory BP. Stratified by the underlying cardiovascular risk, the rate of cardiovascular events in white-coat hypertensive patients and matched normotensive controls were not substantially different. The observation that masked hypertension carries a high cardiovascular risk was replicated in Nigerian Blacks, using home BP monitoring. The thresholds for 24-h mean arterial pressure, i.e., the BP component measured by oscillometric devices, delineating normotension, elevated BP and hypertension were <90, 90 to 92 and ≥92 mmHg. At young age, the absolute risk associated with out-of-office BP was low, but the relative risk was high, whereas with advancing age, the relative risk decreased and the absolute risk increased. Using pulse pressure as an exemplary case, the relative risks of death, cardiovascular endpoints and stroke decreased over 3-fold from 55 to 75 years of age, whereas in contrast absolute risk rose 3-fold. In conclusion, IDACO/IDHOCO forcefully support the notion that the pressing need to curb the hypertension pandemic cannot be met without out-of-the-office BP monitoring.

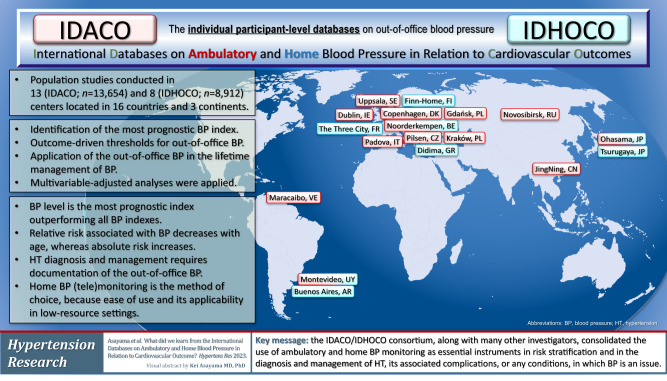

## Introduction

Multiple population studies across the globe highlighted that hypertension is the major modifiable driver of cardiovascular complications [1–3]. According to the 2019 Global Burden of Disease Study, hypertension worldwide and across all ages firmly remains the leading risk factor for death and disability [4]. Accurate measurement is a prerequisite for the management of any risk factor. Blood pressure (BP) not only varies with each heartbeat, but is also influenced by a large number of genetic, endogenous, behavioral, and environmental factors [5]. Given that a large number of measurement is required to come to grips with the diurnal and long-term BP variability, practice guidelines for the management of hypertension unanimously posit that out-of-office BP monitoring is an important clinical asset [6–8].

To assess how out-of-office BP contributes to risk stratification and the management of hypertension, an international consortium set up the International Databases on Ambulatory (IDACO) [9] and Home (IDHOCO) [10] Blood Pressure in Relation to Cardiovascular Outcome. Thorough harmonization, stringent quality control and when available updates ensured that this data resource represents a powerful instrument to assess the relevance of out-of-office BP in a wide array of circumstances, as previously done for office BP as a predictor of cardiovascular mortality and morbidity [1–3]. A previous article summarized the IDACO and IDHOCO findings published prior to 2016 [11]. The present review provides an overview of more recent publications and highlights novel insights acquired over and beyond earlier publications [11].

## The IDACO/IDHOCO databases

As outlined in the protocol articles, cohort studies that were eligible to be included in the IDACO [9] or IDHOCO [10] database were performed in a random population sample or in a group of people representative of the background population. Only two cohorts were indirectly representative of the target population, i.e., the Allied Irish Bank study in IDACO (Supplementary Table 1) [12], and people undergoing a health check at the Hospital Italiano, Buenos Aires, Argentina in IDHOCO (Supplementary Table 2) [10, 13]. Follow-up of all IDACO and IDHOCO cohorts included fatal and nonfatal adverse health outcomes. In addition, studies only qualified for inclusion in the database, if they had been ethically approved by the local competent Institutional Review Board, if at enrolment and follow-up participants had given or renewed informed consent, and if individual studies had articles published in peer-reviewed journals. Whenever required by national regulations, ethical approval was also obtained for the secondary use of anonymized data. The IDACO database was constructed in 2007 [9] and the IDHOCO database in 2012 [10], but whenever possible for the participating centers the outcome data were updated at approximately 5-year intervals. Both databases are a powerful resource allowing individual participant meta-analyses of population cohorts, which in accuracy and statistical power surpass meta-analyses combining summary statistics of various studies [14].

Office BP was measured by a standard mercury sphygmomanometer or a validated automated device, using the appropriate cuff size, after the patients have rested in the sitting or supine position for at least 2 to 5 minutes (details available in the protocol articles [9, 10]). The average of the first two office BP readings was used for analyses. The number of ambulatory BP readings over 24 hours (Supplementary Table 3) and during day- and nighttime (Supplementary Table 4) in IDACO and the number of home BP readings in IDHOCO are listed in the online Data Supplement. All devices for ambulatory (Supplementary Table 3) or home (Supplementary Table 5) BP monitoring applied an oscillometric technique and had passed validation. The IDACO and IDHOCO findings can therefore only be extrapolated to literature data collected with devices validated according to international standardized protocols [15]. Unfortunately, a 2018 review of available devices indicated that of approximately 3000 devices on the market merely 15% had passed validation [16], while a 2021 position paper on BP measurement postulated that only 10% of 4000 had been properly validated [17].

The characteristics of IDACO and IDHOCO participants are summarized in Supplementary Table 6. In both databases, the sex ratio was close to 1:1. Mean age was 51.7 years in IDACO and 59.0 years in IDHOCO. At baseline, the prevalence of smoking, regular alcohol intake, obesity, antihypertensive drug treatment and diabetes was 27.6%, 47.1%, 14.2%, 18.3% and 6.6% among 12,624 IDACO participants and 20.3%, 42.7%, 14.8%, 26.2% and 8.0% among 6887 IDHOCO participants. Office systolic/diastolic BP averaged 131.9/79.7 mmHg in IDACO and 134.3/79.6 mmHg in IDHOCO. The 24-h BP averaged 123.9/74.0 in IDACO and the home BP 127.3/76.2 mmHg in IDHOCO.

## Identification of the most prognostic BP index

Ambulatory BP monitoring substantially refines the risk stratification provided by office BP [17]. The greater number of readings, the absence of digit preference and observer bias, and the reduction of the white-coat effect all contribute to the predictive superiority of ambulatory over office BP [18]. More recently [19], in-office BP readings obtained by automated machines in the absence of an observer were introduced as an alternative to ambulatory monitoring; however, the strength of its association with a cardiovascular outcome is uncertain. Furthermore, the strength of the association of adverse health outcomes with daytime *vs* nighttime BP or with the night-to-day BP ratio (continuously distributed variable) or dipping status (categorical variable) remains debated.

### IDACO findings

Given the uncertainty left by previous studies, a comprehensive analysis of the IDACO database was undertaken to evaluate various types of BP measurements and to assess the strength of their associations with mortality and adverse cardiovascular outcomes [20]. BP was measured by an observer, an automated machine or monitored over 24 h. In this study, automated office BP was the average of the ambulatory recordings during the first recording hour, when the monitors were applied in a medical environment [20]. The BP indexes investigated included: office BP measured by an observer, automated office BP as described above, the 24-h day- and nighttime ambulatory BP, the night-to-day BP ratio, and dipping status. The dipping ratios were 0.80 or less for extreme dipping, more than 0.80 to 0.90 or less for normal dipping, more than 0.90 to 1.00 or less for nondipping, and more than 1.00 for rising (reverse dipping) [18].

**Fig. 1:**
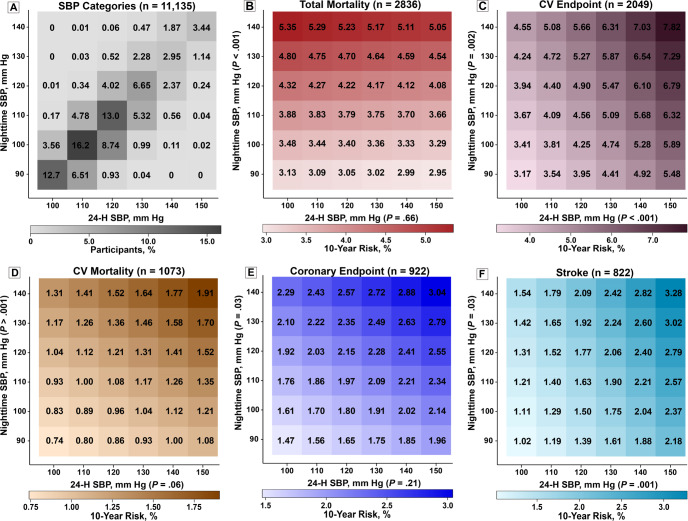
Heat maps depicting 10-year risk in relation to 24-h and nighttime systolic BP in 11,135 study participants. Heat maps were derived by Cox proportional hazards regression with 24-h and nighttime systolic blood pressure (SBP) analyzed as continuous variables. Estimates of 10-year risk were standardized to the average of the distributions in the whole study population (mean or ratio) of cohort identifier, sex, age, body mass index, smoking and drinking, antihypertensive drug treatment, serum cholesterol, history of cardiovascular (CV) disease and diabetes mellitus. Numbers in the grids in **A** represent the percent of participants within each SBP cross-classification category. Numbers in the colored grids (**B**–**F**) represent the 10-year risk of an endpoint. Along the vertical axis, the risks of all endpoints (**B**–**F**) were significantly greater with higher nighttime SBP (*P* ≤ 0.03), but along the horizontal axis only the risk of the composite CV endpoint (**C**; *P* < 0.001) and stroke (**F**; *P* = 0.001) was significantly greater with higher 24-h SBP. Risk of total mortality (**B**), CV mortality (**D**) and a coronary endpoint (**E**) was not significantly associated with 24-h SBP (*P* ≥ 0.06). Reproduced from reference [20], which was published was an open-access article under the terms of the Creative Commons Attribution Non-Commercial-NoDerivs License

Multivariable-adjusted hazard ratios (HRs) expressed the risk of death or a cardiovascular event associated with BP increments of 20/10 mmHg. The composite cardiovascular endpoint included cardiovascular mortality combined with nonfatal coronary events, heart failure, and stroke. Improvement in model performance was assessed by the change in the area under the curve (AUC). Among 11,135 participants enrolled in IDACO (median age, 54.7 years, 49.3% women), 2836 participants died (18.5 per 1000 person-years) and 2049 (13.4 per 1000 person-years) experienced a cardiovascular endpoint. The median follow-up was 13.8 years (5th-95th percentile interval: 2.5-25.1 years). Both endpoints were significantly associated with all single systolic BP indexes (*P* < 0.001). For nighttime systolic BP level, the HR for total mortality was 1.23 (95% confidence interval [CI]: 1.17–1.28) and 1.36 (CI: 1.30–1.43) for the cardiovascular endpoint. For the 24-h systolic BP, the HR for total mortality was 1.22 (CI: 1.16–1.28) and 1.45 (CI: 1.37–1.54) for the cardiovascular endpoint. With adjustment for any of the other systolic BP indexes, the associations of nighttime and 24-h systolic BP with mortality and the composite cardiovascular endpoint remained statistically significant (HRs ranging from 1.17 [CI: 1.10–1.25] to 1.87 [CI: 1.62–2.16]). Heat maps for systolic BP (Fig. 1) showed that along the vertical axis the 10-year risks of all endpoints were significantly greater with higher nighttime systolic BP (*P* ≤ 0.03); whereas, along the horizontal axis, the prognostic impact of 24-h systolic BP was relatively weak, and not significant for the total mortality (*P* = 0.66). Base models that included single systolic BP indexes yielded an AUC of 0.83 for mortality and 0.84 for cardiovascular outcomes. Adding 24-h or nighttime systolic BP to base models that included other BP indexes resulted in incremental improvements in the AUC of 0.0013 to 0.0027 for mortality and 0.0031 to 0.0075 for the composite cardiovascular outcome. Conversely, adding any systolic BP index to models already including nighttime or 24-h systolic BP did not significantly improve model performance. These findings were consistent for diastolic BP. In conclusion, in this population-based IDACO cohort study, higher 24-h and nighttime BP measurements were significantly associated with higher risks of death and the composite cardiovascular outcome, even after adjusting for other office-based or ambulatory BP measurements. Thus, 24-h and nighttime BP may be considered optimal measurements for estimating cardiovascular risk, although statistically, the model improvement compared with other BP indexes was small [20]. A previous IDACO publication clarified that isolated daytime hypertension and isolated nighttime hypertension both predicted adverse cardiovascular health outcomes [21].

### Interpretation within the context of the literature

The IDACO study confirmed previous research, indicating that ambulatory BP monitoring over and beyond measures taken in clinicians’ offices improved risk stratification among patients with [22, 23] or those suspected of having hypertension [24]. It strengthened the notion that nighttime BP measures carry valuable prognostic information [22–24]. A meta-analysis of both summary statistics and individual-level data, combined studies involving patients with hypertension (*N* = 23,856) separately from those of individuals randomly recruited from populations (*N* = 9641) [25]. In both patients and populations, in analyses in which nighttime BP was additionally adjusted for daytime BP, and vice versa, nighttime BP was a stronger predictor than daytime BP [25]. With adjustment for the 24-h BP, both the dipping ratio and dipping status remained significantly associated with outcome, but as evidenced by the generalized R2 statistic and in line with the current findings, added less than 0.6% to the model fit over and beyond the 24-h BP readings [25]. Poor reproducibility of the dipping status, intermediate reproducibility of the dipping ratio, and high reproducibility of the nighttime BP might explain the significantly higher predictive value of the nighttime BP [26]. These findings were to be expected, because the reproducibility of a variable derived from two variables is affected by the variability of both. Possible explanations for the accuracy of the nighttime BP include minimization of confounding by antihypertensive drug treatment that is usually taken in the morning, the standardized conditions during sleep (supine position and absence of movement), and the prognostic value of the basal BP in sedated conditions [27].

## Outcome-driven thresholds

The relation between cardiovascular complications and BP is continuous at least down to an office BP level of 115 mmHg systolic or 75 mmHg diastolic [1]. The continuous nature of the relation with BP not only holds true in hypertensive patients, but in normotensive people as well, so that for instance of all strokes, three-fourths occur in individuals with normal BP on office BP measurement and only one-fourth in patients with office hypertension [1]. The epidemiological evidence does not reveal a sudden increase in the cardiovascular complications associated with BP at the thresholds proposed in the guidelines. However, clinicians need operational thresholds to diagnose hypertension and initiate or adjust antihypertensive drug treatment.

### Systolic and diastolic out-of-office BP

Previous IDHOCO and IDACO analyses produced outcome-driven thresholds for the home [28] and ambulatory [29] BP, corresponding resulting in 10-year cardiovascular risks similar to those associated with optimal (120/80 mmHg), normal (130/85 mmHg), and high (140/90 mmHg) BP on office measurement (Table 1). However, the 2017 American College of Cardiology/American Heart Association guideline reclassified office BP and revised the thresholds for the ambulatory and home BP [6]. Thus, the outcome-driven thresholds for ambulatory BP were recalculated, using the same methods as published before [28, 29]. In short, multivariable-adjusted 10-year risks similar to those associated with elevated office *BP* (120/80 mmHg) and stages 1 and 2 of office hypertension (130/80 mmHg and 140/90 mmHg, respectively) were computed using a bootstrap procedure and random resampling of the whole cohort with replacement. The so-derived thresholds were rounded to the nearest integer value ending in zero or 5 [30].

**Table 1:** Outcome-driven thresholds for ambulatory and home BP derived by IDACO/IDHOCO analyses

OBP category used as reference to compute equivalent 10-year risk	OBP (mmHg)	Home BP (mmHg)	24 h (mmHg)	Day (mmHg)	Night (mmHg)
Reference		[28]	[29]	[29]	[29]
Normal BP	120/80	120/75	115/75	120/80	100/65
Prehypertension	130/85	125/80	125/75	130/85	110/70
Hypertension	140/90	130/85	130/80	140/85	120/70
Severe hypertension	160/100	145/90	–	–	–
Reference			[30]	[30]	[30]
Elevated BP	120/80	–	120/75	120/80	105/65
Stage-1 hypertension	130/80	–	125/75	130/80	110/65
Stage-2 hypertension	140/90	–	130/80	135/85	120/70
Severe hypertension	160/100	–	145/85	150/90	130/80

OBP refers to the office blood pressure thresholds for which the 10-year equivalent risk was computed. For the out-of-office BP thresholds derived in references [28, 29], the OBP thresholds were extracted from the Report of the Joint National Committee on Prevention, Detection, Evaluation, and Treatment of High Blood Pressure (Hypertension 2003: 1206–1252), the Japanese Society of Hypertension (Hypertens Res 2009; 32: 3–107) and the European Societies of Cardiology and Hypertension (J Hypertens 2007: 25: 1105–1187). For the ambulatory BP thresholds derived in reference [30], the OBP levels were those published by the American College of Cardiology/American Heart Association (Circulation 2018; 138: e426-e483). An ellipsis indicates that the out-of-office BP was not computed

*ACC/AHA* American College of Cardiology/American Heart Association, *IDACO* International Database on Ambulatory Blood Pressure in Relation to Cardiovascular Outcome, *IDHOCO* International Database of Home Blood Pressure in Relation to Cardiovascular Outcome

The analyzed IDACO cohort consisted of 11,152 participants recruited from 13 populations [30]. Over 13.9 years (median), 2728 people died, 1033 from cardiovascular disease. Furthermore, 1988 participants experienced a composite cardiovascular endpoint, 893 a coronary event, and 795 a stroke. Using a composite cardiovascular endpoint, systolic/diastolic outcome-driven thresholds indicating elevated 24-h, daytime, and nighttime BP were 117.9/75.2 mmHg, 121.4/79.6 mmHg, and 105.3/66.2 mmHg. For stages 1 and 2 of ambulatory hypertension, the thresholds were 123.3/75.2 and 128.7/80.7 mmHg for the 24-h BP, 128.5/79.6 and 135.6/87.1 mmHg for the daytime BP, and 111.7/66.2 mmHg and 118.1/72.5 mmHg for the nighttime BP. The thresholds for the other study endpoints were similar. After rounding, approximate thresholds for elevated 24-h, daytime, and nighttime BP were 120/75 mmHg, 120/80 mmHg, and 105/65 mmHg. For stages 1 and 2 of hypertension on 24-h, daytime and nighttime BP monitoring, the rounded thresholds were 125/75 and 130/80 mmHg, 130/80 and 135/85 mmHg, and 110/65 and 120/70 mmHg, respectively [30]. The outcome-driven thresholds corresponding to elevated BP and stages 1 and 2 of ambulatory hypertension were similar to those proposed by the 2017 American guideline [6].

Using thresholds proposed in the guidelines, patients can be cross-classified on the basis of the office and out-of-office BP in those with concordant normotension (true normotension) or concordant hypertension (sustained hypertension) on both office and out-of-office BP monitoring. Patients with white-coat hypertension (WCH) have an elevated office BP, but normal out-of-office BP, whereas the opposite is the case in patients with masked hypertension.

### White-coat hypertension

The clinical significance of WCH remains among the fieriest controversies in the management of hypertension [31], with protagonists supporting elevated health hazards risk associated with WCH [32, 33] and other investigators demonstrating little additional risk associated with WCH over and beyond true normotension [34, 35]. In a Taiwanese study [36], the hazard ratios associated with white-coat hypertension *vs* normotension were 1.30 (CI: 0.81–2.09) and 5.59 (CI: 1.22–25.6) for total and cardiovascular mortality, respectively. The Taiwanese study was underpowered to assess cardiovascular mortality, resulting in extremely wide CIs [36], and as the Pressioni Arteriose Monitorate E Loro Associazioni (PAMELA) study [32, 33] did not account for the incidence of nonfatal cardiovascular events. In contrast, the individual participant-level meta-analyses provided unprecedented statistical power and using stringent definitions of white-coat hypertension did not demonstrate a substantially enhance risk of white-coat hypertension compared with normotension [37, 38].

The root cause underlying the debate is the loose definition of WCH in most publications. In untreated participants with mild hypertension enrolled in the Hypertension and Ambulatory Recording Venetia Study (HARVEST) or the Progetto Ipertensione Umbria Monitoraggio Ambulatoriale (PIUMA), white-coat hypertension was most frequent among women, nonsmokers, and individuals with low clinic BP and smaller left ventricular mass [39]. Age stands out as the most important determinant of WCH, while antihypertensive treatment status, risk factors other than BP, and the presence of target organ damage at baseline are major confounders in cohort studies of WCH. To demonstrate the influence of age and sex, in a subject-level meta-analysis, 9550 IDACO participants not taking any antihypertensive medications were combined with 2011 untreated individuals enrolled in Genetic and Phenotypic Determinants of Blood Pressure and Other Cardiovascular Risk Factors (GAPP) [40]. Among individuals aged 18–30, 30–40, and 40-50 years, the average daytime BP was higher than the corresponding office BP. The differences averaged 6.0, 5.2, and 4.7 mmHg systolic, and 2.5, 2.7, and 1.7 mmHg diastolic BP. In contrast, in people aged 60–70 years and ≥70 years, the daytime BP was lower than office BP with differences of 5.0 and 13.1 mmHg systolic, and 2.0 and 4.2 mmHg diastolic [40]. Consequently, the prevalence of WCH exponentially increased from 2.2% to 19.5% from age 18 to 30 to ≥70 years, with negligible differences between women and men [40]. Along similar lines, in untreated participants enrolled in the SKIPOGH study (Swiss Kidney Project of Genes in Hypertension), older age was the sole determinant of white-coat hypertension [41].

Another potential weakness of a large number of articles [32, 33] and meta-analyses dealing with WCH [42] is the use of total or cardiovascular mortality as a primary endpoint. Total and cardiovascular mortality are endpoints, easily obtainable from population registries, but since the introduction of invasive therapies in vascular diseases, such as coronary artery bypass grafting or percutaneous procedures in various vascular beds, carry incomplete outcome information. Therefore, fatal combined with nonfatal cardiovascular complications should be considered as the endpoints of choice.

**Fig. 2:**
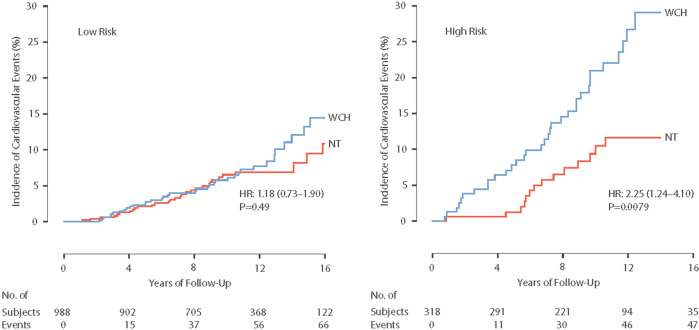
The analysis is broken down according to low (left, *N* = 494 in both white-coat hypertension [WCH] and normotensive [NT] groups) and high (right, *N* = 159 in both groups) cardiovascular disease (CVD) risk. The number of incident CVD events in the WCH WCH and NT groups totaled 37 and 32 in the low-risk group and 33 and 16 in the high-risk group, respectively. The numbers below the horizontal axes are the number of subjects experiencing a CVD event and the number of subjects still in follow-up at 4-year intervals. HR is the unadjusted hazard ratio estimating the relative event rate in subjects with WCH versus the normotensive participants. The HR tended to be higher in the WCH group compared with the normotensive group (interaction *P*-value, 0.074). Reproduced with permission from reference [38]

Using 11-cohort population-based IDACO data, daytime ambulatory BP and office BP were recorded in 653 untreated study participants with WCH and 653 normotensive control subjects [43]. The cut-off limits for office and daytime ambulatory BP were 140/90 mmHg and 135/85 mmHg, respectively. The contemporary European Society Hypertension guideline was applied to develop a 5-stage risk score [44]. Low risk was defined as 0 to 2 risk factors, and high risk as ≥3 to 5 risk factors, diabetes, and/or history of prior CVD events. Age- and cohort-matching was done between the 653 untreated participants with WCH and the 653 normotensive control participants. Over a median 10.6-year follow-up (5th-95th percentile interval: 2.5–18.1 years), the incidence of new fatal combined with nonfatal cardiovascular endpoints was higher in 159 high-risk subjects with WCH compared with 159 cohort- and age-matched high-risk normotensive individuals (Fig. 2). With the multivariable adjustment, the HR in patients with high-risk WCH *vs* high-risk normotensive patients was 2.06 (CI: 1.10–3.84; *P* = 0.0079). The HR was not significant for 494 participants with low-risk WCH and age-matched low-risk normotensive participants (HR: 1.06; CI: 0.66–1.72; *P* = 0.80). Subgroup analysis by age showed that the association between WCH and incident cardiovascular events was limited to older (≥60 years) high-risk WCH subjects, i.e., the adjusted HR was 2.19 (CI: 1.09–4.37; *P* = 0.027) in the older high-risk group and 0.88 (CI: 0.51–1.53; *P* = 0.66) in the older low-risk group (interaction *P* = 0.016). In summary, at least in this IDACO analysis [43], stratified for the underlying cardiovascular risk, the risk of incident cardiovascular endpoints in most patients with WCH was not substantially higher than in normotensive control individuals. These findings were concordant with an earlier IDACO article [37].

Recent hypertension guidelines acknowledge no solid evidence that antihypertensive treatment reduces adverse health outcomes in WCH patients [6–8]. In view of the effects of aging on BP, regular follow-up of the office and out-of-office BP is a recommended approach in WCH patients. Moreover, over and beyond the BP level, risk stratification is common practice in the decision to start antihypertensive treatment [6–8], so that WCH patients at high risk or with comorbidities are eligible for BP lowering treatment.

### Masked hypertension

In contrast to controversy about WCH, there is a large consensus among hypertension specialists that masked hypertension carries a risk similar to or only slightly less than sustained hypertension [45]. Of 7030 participants included in one of the initial IDACO analyses [37], using as thresholds for office and daytime ambulatory hypertension levels of 140/90 mmHg and 135/85 mmHg, 1024 patients (14.6%) had masked hypertension and 1790 (25.5%) sustained hypertension. Over a median follow-up of 9.5 years (5th-95th percentile interval: 2.7–14.0 years), the composite cardiovascular endpoint occurred in 140 patients with masked hypertension (13.7%) and in 403 (22.5%) with sustained hypertension, resulting in multivariable-adjusted HR of 1.62 (CI: 1.35-1.96; *P* < 0.0001) and 1.80 (CI: 1.59–2.03; *P* < 0.0001), respectively.

Observing target organ damage in patients with optimal or normal office BP represents a major clue suggesting that masked hypertension might be present. Signs of target organ damage include hypertensive retinopathy, left ventricular hypertrophy, diastolic or systolic left ventricular dysfunction, reduced glomerular filtration rate, microalbuminuria, or a history of cardiovascular disease. In previous publications [46, 47], we identified various other risk factors associated with a high probability of masked hypertension diagnosed either by self-measurement of BP at home [47] or ambulatory BP monitoring [46]. In IDHOCO [47], participants with masked hypertension according to the 135/85-mmHg threshold, compared with participants with true optimal, normal, or high-normal BP, were more likely to be men (52.6% vs 37.1%), to smoke (28.7% vs 22.6%), to have diabetes (13.0% vs 5.2%) or a history of cardiovascular disease (14.6% vs 6.4%), and to be older (62.3 vs 53.4 years) and more obese (27.0 vs 24.6 kg/m^2^). In IDACO [46], using a daytime systolic/diastolic BP of 135/85 mmHg, the risk factors associated with masked hypertension were similar.

Masked hypertension is a forerunner of sustained hypertension [48, 49]. However, expert opinion on the reproducibility of masked hypertension remains divided. In a Chinese study [50], daytime BP thresholds for masked hypertension were met in 5 patients (11.1%) for systolic BP, in 25 (55.6%) for diastolic BP and in 15 (33.3%) for both. Among these 45 patients, over 4 weeks of follow-up, masked hypertension remained present in 28 (62.2%;CI: 48.1–76.3%), whereas 13 (28.9%; 15.7–42.1%) and 4 (8.9%; 0.6–17.2%) converted to normotension (daytime BP < 135/85 mmHg) or sustained hypertension (office BP ≥ 140/90 mmHg), respectively [50]. Substituting daytime by 24-hour BP, using 130/80 mm Hg as the threshold, produced consistent results [50]. In an American study [51], the prevalence of masked hypertension was assessed at 2 visits 1 week apart, using office-daytime ambulatory BP or office-home BP pairings. Using daytime BP, the prevalence of masked hypertension was 54% and 53%, with an agreement of 73% (κ = 0.47; CI: 0.21–0.72). MH was less prevalent (43% and 35%) using HBPM-office pairings, with an agreement of 69% (κ = 0.34; CI: 0.06–0.62). The authors concluded that masked hypertension appears to have fair-to-moderate reproducibility and that home BP monitoring might not be adequate for detecting masked hypertension [51].

The full prognostic significance of masked hypertension was assessed in a study comparing the prevalence and determinants of masked hypertension diagnosed with self-monitored home BP (≥135/85 mmHg) among 293 Nigerians with a reference population consisting of 3615 IDHOCO participants with a similar sex and age distribution [52]. In the reference population, the prevalence of masked hypertension was 14.6% overall and 11.1% and 39.6% in untreated and treated participants, respectively. Among Nigerians, the prevalence standardized to the sex and age distribution of the reference population was similar, with rates of 14.4%, 8.6%, and 34.6%, respectively [52]. The mutually adjusted ORs of having masked hypertension in Nigerians were 2.34 (CI: 1.39–3.94) for a 10-year higher age, 1.92 (CI: 1.11–3.31) and 1.70 (CI: 1.14–2.53) for 10- or 5-mmHg increments in systolic or diastolic office BP, and 3.05 (CI: 1.08–8.55) for being on antihypertensive therapy. The corresponding estimates in the reference population were similar with ORs of 1.80 (CI: 1.62–2.01), 1.64 (CI: 1.45–1.87), 1.13 (CI: 1.05–1.22), and 2.84 (CI: 2.21–3.64), respectively [52]. However, the associations of ECG voltages and voltage-duration products and the risk of electrocardiographic left ventricular hypertrophy with BP were, on average, twice steeper in Black Nigerians compared with a White Flemish reference population [53]. Pro-actively searching for masked hypertension is extremely important for the proper management of patients with high-normal office BP and in office normotensive patients with target organ hypertension or diabetes.

Given the risk associated with masked hypertension, the results of the ANTIhypertensive treatment in MASKed hypertension for target organ protection (ANTI-MASK) trial are eagerly awaited. ANTI-MASK (NCT02893358) is a multicenter, randomized, double-blind, placebo-controlled clinical trial [54]. Eligible patients are 30 to 70 years old who have untreated masked hypertension and at least one sign of target organ damage, including electrocardiographically diagnosed left ventricular hypertrophy, brachial-ankle pulse wave velocity ≥1400 cm/s, or a random urinary albumin-to-creatinine ratio ≥2.5 mg/mmol in men and ≥3.5 mg/mmol in women. In this first trial of the management of masked hypertension, Chinese patients are randomized to control or BP lowering treatment, using ambulatory BP as a guide. Treatment in the active arm consists of allisartan with the possible addition of amlodipine. The primary endpoint is the improvement rate of the progression of target organ damage assessed after 12 months of follow-up. Recruitment of the required 320 randomized patients was completed in 2021.

### Mean arterial pressure

Mercury is being phased out. The oscillometric method is therefore becoming dominant to the auscultatory Korotkoff approach in use since 1910 [55]. The proprietary software implemented in automated oscillometric devices draws an envelope around the pressure oscillations in the brachial cuff and estimates mean arterial pressure (MAP) as the cuff pressure at the point of maximal oscillations [56, 57]. From the so estimated MAP, the software then computes systolic and diastolic BP [56, 57]. For validated devices, the fault tolerance around the calculated systolic and diastolic BP is ±5 mmHg [58]. Furthermore, MAP is similar throughout the arterial tree [59], thereby avoiding the dilemma as to whether central compared with brachial BP confers higher cardiovascular risk [60]. In addition, MAP captures risk-related information associated with both systolic and diastolic BP [61]. In an individual participant meta-analysis of 1 million people, office MAP was a better predictor of vascular mortality than systolic or diastolic BP [3].

**Fig. 3:**
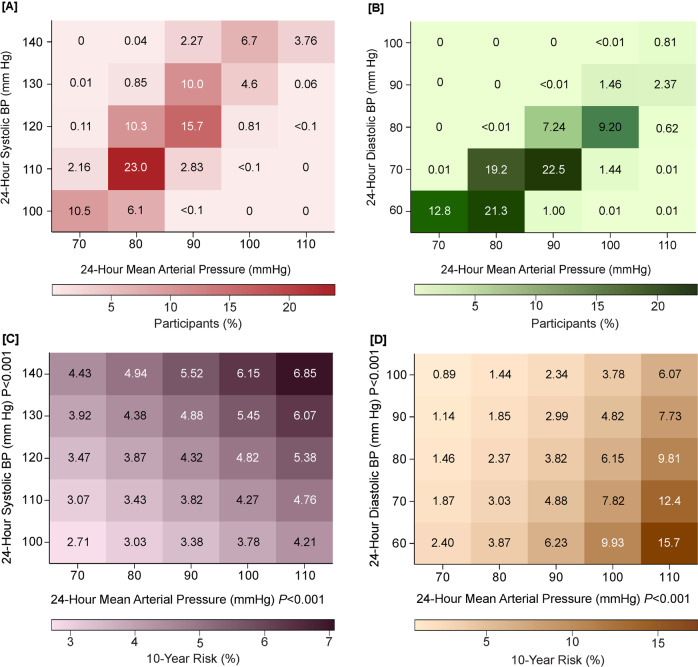
Heat maps depicting the 10-year risk of a composite cardiovascular endpoint in relation to 24-h mean arterial pressure (MAP), systolic and diastolic BP in 11,596 IDACO participants. Numbers in the **A** and **B** grids represent the percentage of participants within each BP cross-classification category; numbers in **C** and **D** represent the 10-year risks. Heat maps were derived by Cox proportional hazards regression with systolic BP (**C**) or diastolic BP (**D**) plotted along the vertical axis and MAP along the horizontal axis. Estimates of the 10-year risk were adjusted for cohort (random effect), sex, and baseline characteristics including age, body mass index, smoking and drinking, serum cholesterol, antihypertensive drug intake, history of cardiovascular disease, and diabetes. Higher MAP consistently conferred greater risk (*P* < 0.001) with an additional contribution of systolic BP (*P* < 0.001 (**C**)), whereas higher diastolic BP attenuated the risk (*P* < 0.001 (**D**)). Reproduced from reference [62], which was published was an open-access article under the terms of the Creative Commons Attribution Non-Commercial-NoDerivs License

Hypertension guidelines do not propose how MAP, the BP level which is actually measured by most currently marketed BP monitors, might be used in risk stratification [6–8]. Given the clinical underuse of MAP and the predictive superiority of 24-h BP [20], the IDACO cohort was analyzed to derive outcome-driven thresholds for 24-h MAP, which might be useful in clinical practice, based on the strength of the association of MAP with fatal and nonfatal cardiovascular endpoints [62]. Twenty-four-hour MAP levels of <90 (normotension, *N* = 6183), 90 to <92 (elevated MAP, *N* = 909), 92 to <96 (stage-1 hypertension, *N* = 1544), and ≥96 (stage-2 hypertension, *N* = 2960) mmHg yielded 10-year risks of experiencing a major cardiovascular endpoint, equivalent to the 2017 American thresholds for office systolic and diastolic BP [6]. Compared with 24-h MAP normotension, HRs were 0.96 (CI: 0.80–1.16), 1.32 (CI: 1.15–1.51), and 1.77 (CI: 1.59–1.97), for elevated and stage-1 and stage-2 hypertensive MAP. On top of 24-h MAP, higher 24-h systolic BP increased, whereas higher 24-h diastolic BP attenuated risk (*P* < 0.001; Fig. 3).

From a physiological point of view, BP and blood flow can be broken down into a pulsatile component with systolic and diastolic BP representing the extremes of the BP oscillations around MAP, which drives organ perfusion [63, 64]. When peripheral resistance increases by rarefaction or remodeling of arterioles, MAP rises with parallel increments in systolic and diastolic BP. However, when there is an additional reduction of arterial compliance, as occurs with stiffening of the large arteries, both systolic BP and MAP increase, whereas diastolic BP decreases [65]. Figure 3 illustrates these concepts, showing that the 10-year risk of the primary endpoint was consistently greater with higher MAP with an additional contribution of systolic BP, whereas higher diastolic BP attenuated the risk.

These IDACO findings are in keeping with the concepts generated by the Framingham Heart Study based on office BP [66]. More specifically, in the general population, starting from middle-age, there is a gradual shift from the steady BP component (diastolic BP) to the pulsatile component (systolic SBP or pulse pressure [PP]) as predictors of coronary heart disease [66]. In the Physicians’ Health Study [67], cardiovascular disease was predicted by systolic and diastolic BP and their linear combination—MAP—in younger men (<60 years), whereas in older men (≥60 years) systolic BP and PP were the main drivers of cardiovascular risk. From a lifecourse perspective, IDACO analyses demonstrated that MAP is a risk factor across the age range [62], whereas PP is only a weak risk factor in the elderly [68, 69]. Using oscillometric devices for 24-h BP monitoring, <90, 90 to <92, 92 to <96, and ≥96 mmHg are the thresholds for 24-h MAP, delineating normotension, elevated BP and stage-1, and stage-2 hypertension. Below 60 years of age, PP does not carry any risk [68]. In the elderly (≥60 years), the 24-h ambulatory PP is a stronger predictor than PP derived from office BP [68]. The 24-h ambulatory PP threshold signifying increased cardiovascular risk was as high as ≥68.1 mmHg (top decile of the PP distribution), but such high PP contributed only 0.3% to the overall cardiovascular in elderly over and beyond other risk factors [68]. How the BP associated risk changes over the human life span is addressed in the next section with ambulatory PP as the exemplary BP component.

## A lifetime perspective

The Bogalusa Heart Study [70, 71] and other cohort studies [72] demonstrated that hypertension and associated risk factors and comorbidities originate in childhood and young adulthood. Tracking indicated that individuals keep their position in the distribution of a risk factor, such as BP; whereas, horse racing refers to the accelerated increase in a risk factor with a higher position of an individual in the risk distribution at a young age [73]. An expert working group therefore called for a life-course approach to the management of hypertension [74].

### Relative vs absolute risk

**Fig. 4:**
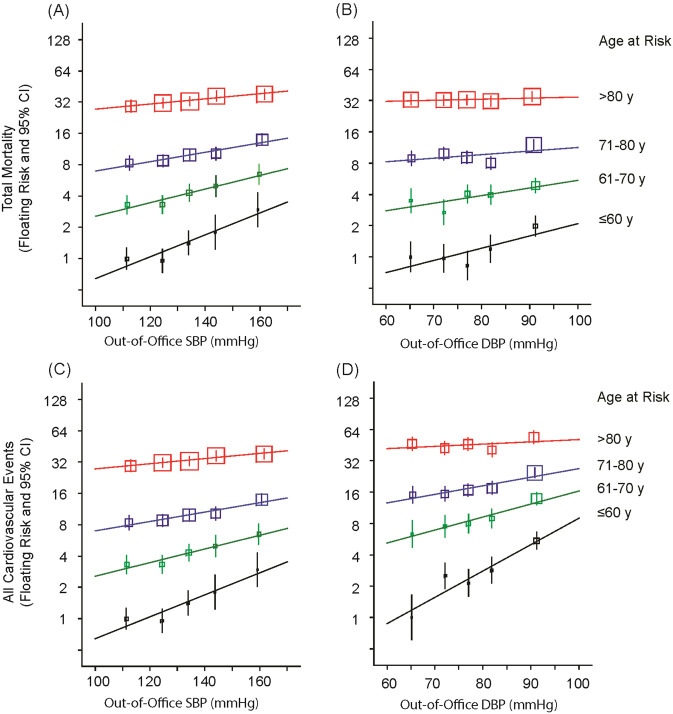
Total mortality (**A**, **B**) and cardiovascular endpoints (**C**, **D**) by age-at-risk groups and categories of out-of-office blood pressure. Point estimates and 95% confidence intervals for the floating absolute risks were plotted along the vertical axis. The size of the squares is proportional to the inverse the variance of each hazard ratio. Risk estimates were derived by Cox regression with the Lexis expansion for age. The analyses were stratified by cohort and adjusted for sex, body mass index, serum cholesterol, smoking and drinking, antihypertensive drug treatment and history of diabetes mellitus and cardiovascular disease. The categories plotted along the horizontal axis are The categories plotted along the horizontal axis are <120, 120–129, 130–139, 140–149 and ≥150 mmHg for systolic blood pressure (SBP) and <70, 70–74, 75–79, 80–84 and ≥85 mmHg for the diastolic blood pressure (DBP). Log-linear relations were fitted for each age group for out-of-office SBP (**A**, **C**), and DBP (**B**, **D**). Reproduced from reference [75], which was published was an open-access article under the terms of the Creative Commons Attribution Non-Commercial-NoDerivs License

**Fig. 5:**
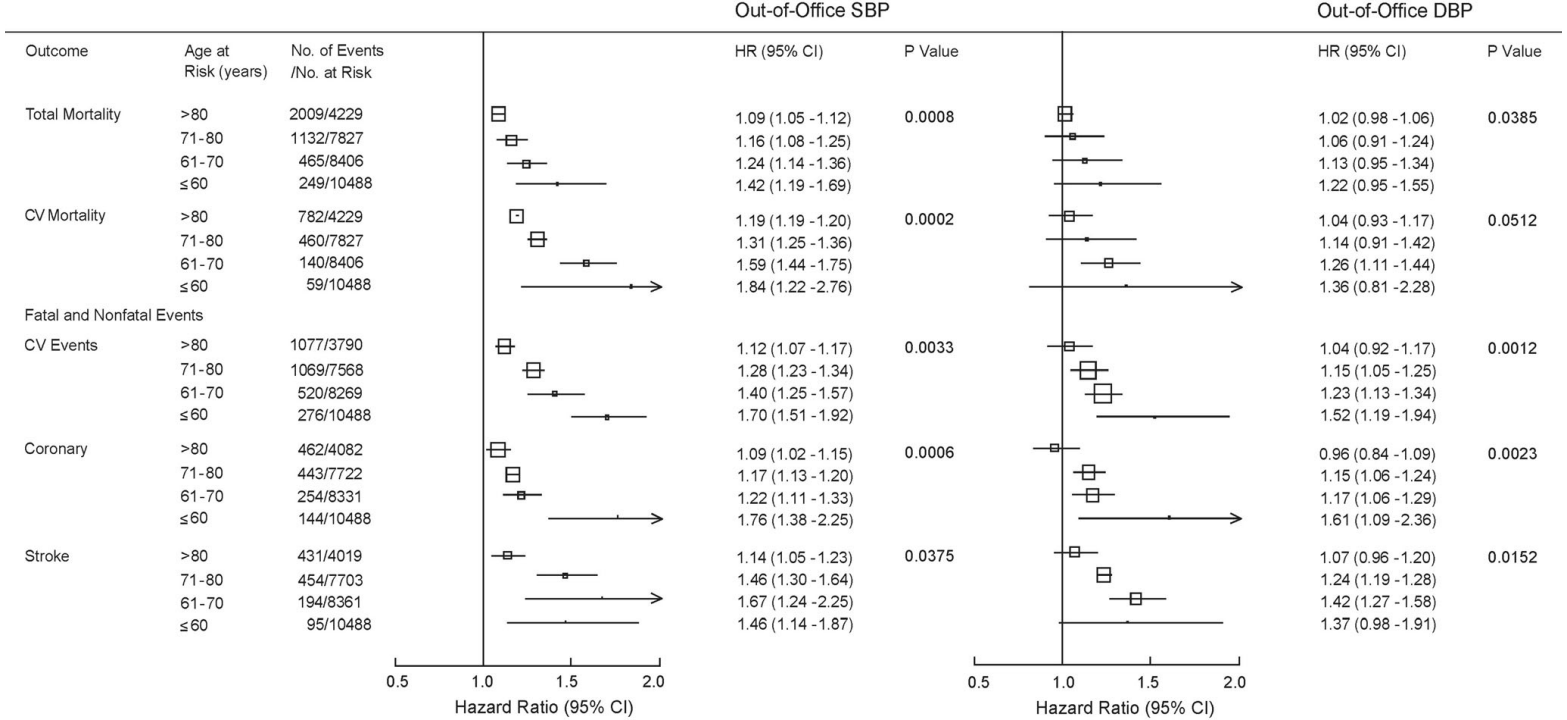
Hazard ratios for out-of-office blood pressure by four age-at-risk groups The Cox models were stratified by cohort and adjusted for sex, age, body mass index, serum cholesterol, smoking and drinking, antihypertensive drug treatment and history of diabetes mellitus and cardiovascular disease. Hazard ratios, given for four age groups, express the risk associated with increments in out-of-office blood pressure (daytime or home) of 20 mmHg systolic (SBP) or 10 mmHg diastolic (DBP). Squares representing the point estimates have a size proportional to the inverse of the variance. Horizontal lines denote the 95% confidence interval. *P*-values are for trend across the four age groups. Reproduced from reference [75], which was published was an open-access article under the terms of the Creative Commons Attribution Non-Commercial-NoDerivs License

Seminal publications addressed the age- sex- and ethnicity-specific relevance of office BP as the determinant of the incidence of mortality and fatal and nonfatal cardiovascular endpoints [1–3], but no such analysis was ever undertaken for out-of-office BP. To address this knowledge gap, we pooled the IDHOCO and IDACO databases [75]. At baseline, daytime ambulatory (*N* = 12,624) or home (*N* = 5297) BP were measured in 17,921 participants (51.3% women; mean age, 54.2 years) from 17 population cohorts. Using multivariable Cox regression, floating absolute risk was computed across 4 age bands (≤60, 61–70, 71–80, and >80 years). Over 236,491 person-years, 3855 people died and 2942 cardiovascular events occurred. From levels as low as 110/65 mmHg, risk log-linearly increased with higher out-of-office systolic/diastolic BP (Fig. 4). From the youngest to the oldest age group, rates expressed per 1000 person-years increased (*P* < 0.001) from 4.4 (CI: 4.0–4.7) to 86.3 (CI: 76.1–96.5) for total mortality and from 4.1 (CI: 3.9–4.6) to 59.8 (CI: 51.0–68.7) for cardiovascular events, whereas hazard ratios per 20-mmHg increment in systolic out-of-office BP decreased (*P* ≤ 0.0033) from 1.42 (CI: 1.19–1.69) to 1.09 (CI: 1.05–1.12) and from 1.70 (CI: 1.51–1.92) to 1.12 (CI: 1.07–1.17), respectively (Fig. 5). These age-related trends were similar for out-of-office diastolic BP and were generally consistent in both sexes and across ethnicities. In conclusion, adverse health outcomes among adults were directly associated with out-of-office BP. At a young age, the absolute risk associated with out-of-office BP was low, but the relative risk was high, whereas with advancing age, the relative risk decreased and the absolute risk increased [75].

### Arterial stiffening

Over the human lifespan, aging and age-related risk factors, such as hypertension, renal dysfunction, and type-2 diabetes, lead to stiffening of the central elastic arteries. Consequently, the systolic load on the arterial walls is cushioned less, a phenomenon further amplified by the early return of reflected waves in early systole, while the tensile force maintaining a continuous blood flow during diastole diminishes [76]. From middle age onwards, PP widens because systolic BP continues to rise until old age, whereas diastolic BP decreases [65]. In HARVEST (*N* = 1141), untreated participants with isolated systolic hypertension on office measurement at 18-45 years of age over 6 years of follow-up had a smaller risk of developing ambulatory hypertension (13.8%) compared with patients with isolated diastolic hypertension (24.8%) or mixed systolic and diastolic hypertension (61.4%) [77].

To analyze the prognostic significance of ambulatory PP, the IDACO cohort was stratified into 4663 young (18–49 years) and 7185 older adults (≥50 years), and brachial PP was recorded over 24 h [69]. In this IDACO report [69], total mortality and a composite of all cardiovascular events were coprimary endpoints. Cardiovascular death, coronary events, and stroke were secondary endpoints. In young adults (median follow-up, 14.1 years; mean PP, 45.1 mmHg), greater PP was not associated with absolute risk; the endpoint rates were ≤2.01 per 1000 person-years. The multivariable-adjusted HRs expressing relative risk per 10-mmHg PP increments were less than unity (*P* ≤ 0.027) for the composite cardiovascular endpoint (0.67; CI: 0.47–0.96) and cardiovascular mortality (0.33; CI: 0.11–0.75). In older adults (median follow-up, 13.1 years; mean PP, 52.7 mmHg), the endpoint rates, expressing absolute risk, ranged from 22.5 to 45.4 per 1000 person-years and the adjusted HRs ratios, reflecting relative risk, from 1.09 to 1.54 (*P* < 0.0001). The PP-related relative risks of death, the composite cardiovascular endpoint, and stroke decreased over 3-fold from age 55 to 75 years, whereas in contrast absolute risk rose 3-fold [69], confirming the concept that relative risk decreases with advancing age, whereas absolute risk increases with higher age [75].

The IDACO findings on PP also are in line with vested pathophysiological concepts. Elastin and collagen are the major constituents of the extracellular matrix in the media of the central elastic arteries. Elastin provides reversible extensibility during systole, while collagen generates the tensile strength of the arterial wall. As people age, the elastic fibers become fragmented and the mechanical load is transferred to collagen fibers, which are up to 1,000 times stiffer than elastin [78]. This process already starts in young adulthood [76], but elastin deposition by vascular smooth muscle cells only occurs during fetal development and early infancy [79, 80] and is switched off thereafter. This implies that elastin fiber damage is basically irreversible. Instead, more collagen is produced, which decreases the elastin-to-collagen ratio and shifts the mechanical arterial properties towards the stiffer range of collagen fibers.

## Conclusions and perspectives

The IDACO/IDHOCO consortium, along with many other investigators, consolidated the application of ambulatory and home BP monitoring as essential instruments in risk stratification and in the diagnosis and management of hypertension, its associated complications, or any condition, in which BP is an issue, such as heart failure or chronic kidney disease.

From a clinical perspective, the outcome-driven thresholds generated for the ambulatory and home BP can be applied in both sexes and across the adults age range up to 80 years of age [75, 81], while in the very elderly overtreatment should be avoided [82]. Ambulatory BP monitoring is the state-of-the-art method, but requires expensive equipment and trained observers; therefore, this approach can only be applied in expert hypertension centers. Given that the BP level is closest associated with adverse health outcomes [20] and that ambulatory and home BP monitoring perform similarly in assessing the BP-related risk, BP self-measurement at home is the technique to be deployed in primary care and in low-resource settings. As an exemplary case, one may extrapolate the finding of a 9.0% prevalence of masked hypertension among untreated Nigerians to ≈50 million Nigerians in the same age bracket diagnosed to be normotensive by clinic measurement [83, 84]. This translates to ≈4.5 million Nigerians living under the burden of undetected hypertension. Home BP monitoring is feasible in these low-resource settings [85].

From a research perspective, long-term telemonitoring of BP is not only feasible, but also well accepted by patients and caregivers [86, 87]. If automated devices are used and guidelines for the timing of the home measurements are followed [17], the recorded home BP levels are free of observer bias and new technologies currently available and validated allow assessment of the BP during sleep. Moreover, in long-term trials, BP telemonitoring is a powerful instrument in educating and empowering patients, increases adherence to antihypertensive drugs, allows detection of symptoms that occur between clinic visits, and reduces the number of clinic visits required for optimizing drug treatment [88, 89]. In conclusion, addressing the worldwide hypertension pandemic—the leading cause of cardiovascular death and living with disability [4]—requires accurate BP measurement for its diagnosis and management [74]. This goal cannot be achieved without out-of-the-office BP monitoring.

## Supplementary information

Supplementary Materials
