# Supplementary material for: What did we learn from the International Databases on Ambulatory and Home Blood Pressure in Relation to Cardiovascular Outcome?
Source: Hypertens Res. 2023 Feb 3;46(4):934–49. doi: 10.1038/s41440-023-01191-4 (PMC10073019; doi:10.1038/s41440-023-01191-4)
Supplement: Supplementary file 1 — Supplementary Materials [file 41440_2023_1191_MOESM1_ESM.doc]

***Hypertension Research***

Supplemental Material to “*What did we learn from the International Databases on Ambulatory and Home Blood Pressure in Relation to Cardiovascular Outcome?*”.

**Table of contents**

Supplementary Table 1 Recruitment and follow-up of IDACO participants by cohort p2

Supplementary Table 2 Recruitment and follow-up of IDHOCO participants by cohort p3

Supplementary Table 3 24-H ambulatory blood pressure monitoring by IDACO cohort p4

Supplementary Table 4 Number of daytime and nighttime blood pressure readings by IDACO cohort p5

Supplementary Table 5 Home blood pressure measurement by IDHOCO cohort p6

Supplementary Table 6 Baseline characteristics of IDACO and IDHOCO participants p7

**Supplementary Table 1**

Recruitment and follow-up of IDACO participants by cohort

| Catchment area | Sampling frame | Recruitment | | Participation  rate (%) | N° of participants | | Median follow-up  in years  (5-95% interval) |
| --- | --- | --- | --- | --- | --- | --- | --- |
|  |  | Time  period  (years) | Invitation |  | In database  (n=13,654) | analyzed  (n=12,624) |  |
| Ohasama, Iwate, Japan | People aged ≥40 years | 1988–1994 | Address list | 78 | 1535 | 1003 | 22.0 (5.7–26.5) |
| JingNing, Zhejiang, China | Family-based random sample | 2003–2008 | All villagers invited | 62 | 895 | 880 | 4.0 (3.5–7.6) |
| Oktyabrsky, Novosibirsk, Russia | Family-based random sample | 1999–2001 | Address list | 68 | 306 | 304 | 16.4 (8.1–17.5) |
| Niepolomice, Kraków, Poland | Family-based random sample | 1999–2008 | Address list | 54 | 413 | 391 | 13.5 (6.1–14.3) |
| Gdańsk, Poland | Family-based random sample | 2008–2010 | Address list | 90 | 215 | 213 | 5.6 (4.7–6.7) |
| Pilsen, Czech Republic | Family-based random sample | 2000–2001 | Address list | 82 | 174 | 174 | 14.1 (13.8–14.4) |
| Padova, Italy | Population-based sample of women and men≥18 years | 1999–2007 | Address list | 73 | 314 | 314 | 13.3 (12.6–14.5) |
| Noordkempen, Belgium | Family-based random sample | 1985–2008 | Address list | 78 | 2904 | 2580 | 18.1 (8.6–25.8) |
| Uppsala, Sweden | Men aged ≥50 years | 1991–1995 | Population census | 73 | 1143 | 1135 | 15.1 (3.5–22.2) |
| Copenhagen County, Denmark | Stratified random sample of women and men aged 30, 40, 50 and 60 years | 1993–1997 | Population registry | 83 | 2311 | 2296 | 16.3 (5.2–17.3) |
| Dublin, Ireland | Bank employees working at branches across Ireland | 1989–1991 | All invited | 14 | 981 | 961 | 17.6 (16.5–18.2) |
| Maracaibo, Venezuela | City resident aged ≥55 years | 1998–2008 | Population census | 71 | 604 | 601 | 8.1 (1.7–13.7) |
| Montevideo, Uruguay | Age-stratified random sample | 1995–1998 | Members of a  health insurance organization | 78 | 1859 | 1772 | 9.0 (4.2–10.7) |

The European Project on Genes in Hypertension included participants recruited in Novosibirsk, Kraków, Gdańsk, Pilsen and Padova. Participants from Padova were recruited in Mirano in the province of Venice and in Torrebelvicino and Valli del Pasubio in the province of Vicenza. Participation rate refers to the percentage of people invited at enrolment, who provided written informed consent and were enrolled.

**Supplementary Table 2**

Recruitment and follow-up of IDHOCO participants by cohort

| Catchment area | Sampling frame | Recruitment | | Participation  rate (%) | N° of participants | | Median follow-up  in years  (5-95% interval) |
| --- | --- | --- | --- | --- | --- | --- | --- |
|  |  | Time  period  (years) | Invitation |  | In database  (n=7571) | Analyzed  (n=6887) |  |
| Ohasama, Iwate, Japan | People aged ≥35 years | 1988–1995 | Address list | 80 | 2758 | 2115 | 20.7 (3.6-27.5) |
| Tsurugaya, Japan | All residents of Tsurugaya  aged ≥ 70 years | 2002 | Address list | 43 | 836 | 817 | 5.5 (2.3–5.6) |
| Noordkempen, Belgium | Family-based random sample | 2012-2013 | Address list | 78 | 411 | 411 | 2.9 (2.1–3.7) |
| Didima, Greece | Residents of Didima  aged ≥18 years | 1997 | Address list | 76 | 665 | 665 | 18.9 (4.2–19.4) |
| Finnish National Sample | Two-stage cluster sample of people aged 45–74 years | 2000–2001 | Population registry | 48 | 2075 | 2074 | 13.2 (6.6–13.3) |
| Buenos Aires, Argentina | Hospital Italiano | 2008-2010 | Referrals for  health check-up | 100 | 426 | 406 | 3.7 (1.7–4.5) |
| Montevideo, Uruguay | Age-stratified random sample | 1996–1998 | Members of a  health insurance organization | 34 | 400 | 399 | 8.9 (5.7–10.6) |

Participation rate refers to the percentage of people invited at enrolment, who provided written informed consent and were enrolled.

**Supplementary Table 3**

24‑H ambulatory blood pressure monitoring by IDACO cohort

| Study cohorts | N° of  people  (n=10,864) | Monitoring device | Minutes between readings | |  | N° of Readings over 24 hours | | | | | | |
| --- | --- | --- | --- | --- | --- | --- | --- | --- | --- | --- | --- | --- |
|  |  |  | Day | Night |  | Programmed | Median | P5 | P25 | P75 | P95 | |
| Ohasama, Iwate, Japan | 1001 | ABP-630, Nippon Colin | 30 | 30 |  | 48 | 46 | 36 | 42 | 48 | 50 |  |
| JingNing, Zhejiang, China | 875 | 90207, SpaceLabs | 20 | 30-45 |  | 59–65 | 56 | 48 | 55 | 57 | 62 |  |
| Oktyabrsky, Novosibirsk, Russia | 300 | 90202, SpaceLabs | 15 | 30 |  | 76 | 71 | 56 | 65 | 75 | 78 |  |
| Niepolomice, Kraków, Poland | 389 | 90202, SpaceLabs | 15 | 30 |  | 76 | 74 | 54 | 63 | 77 | 79 |  |
| Gdańsk, Poland | 212 | TM-2430, A&D | 20 | 45 |  | 65 | 62 | 50 | 59 | 64 | 64 |  |
| Pilsen, Czech Republic | 165 | 90202, SpaceLabs | 20 | 45 |  | 65 | 75 | 56 | 71 | 80 | 82 |  |
| Padova, Italy | 314 | 90202, SpaceLabs | 15 | 30 |  | 76 | 76 | 64 | 74 | 77 | 78 |  |
| Noordkempen, Belgium | 1412 | 90202, SpaceLabs | 20 | 40 |  | 55 | 53 | 38 | 41 | 56 | 58 |  |
| Uppsala, Sweden | 1097 | Accutracker II | 20–30 | 20–60 |  | 41–72 | 66 | 44 | 53 | 75 | 85 |  |
| Copenhagen County, Denmark | 2142 | TM-2421, A&D | 15 | 30 |  | 80 | 80 | 68 | 80 | 81 | 83 |  |
| Dublin, Ireland | 930 | 90202 and 90207, Spacelabs | 30 | 30 |  | 48 | 46 | 39 | 44 | 48 | 49 |  |
| Maracaibo, Venezuela | 589 | 90207, SpaceLabs | 15 | 30 |  | 80 | 67 | 53 | 61 | 71 | 77 |  |
| Montevideo, Uruguay | 1438 | 90207, SpaceLabs | 20 | 40 |  | 60 | 67 | 53 | 61 | 71 | 77 |  |

The TM-2421 and TM-2430 monitors implement both an auscultatory and an oscillometric technique. However, only oscillometric readings were used for analysis. All devices passed validation.

**Supplementary Table 4**

Number of daytime and nighttime blood pressure readings by IDACO cohort

| Study cohorts | Daytime | | | | | | |  | Nighttime | | | | | | |
| --- | --- | --- | --- | --- | --- | --- | --- | --- | --- | --- | --- | --- | --- | --- | --- |
|  | N° of  people  (n=12,624) | Planned  readings | Median | P5 | P25 | P75 | P95 |  | N° of  People  (n=10,864) | Planned  readings | Median | P5 | P25 | P75 | P95 |
| Ohasama, Iwate, Japan | 1003 | 20 | 19 | 14 | 17 | 21 | 22 |  | 1001 | 12 | 11 | 8 | 11 | 12 | 12 |
| Jing-Ning, Zhejiang, China | 880 | 30 | 30 | 21 | 29 | 31 | 32 |  | 875 | 8–12 | 8 | 7 | 8 | 8 | 12 |
| Oktyabrsky, Novosibirsk, Russia | 304 | 40 | 37 | 26 | 33 | 40 | 42 |  | 300 | 12 | 12 | 11 | 12 | 12 | 12 |
| Niepolomice, Kraków, Poland | 391 | 40 | 39 | 26 | 32 | 41 | 43 |  | 389 | 12 | 12 | 8 | 10 | 12 | 12 |
| Gdańsk, Poland | 213 | 30 | 29 | 21 | 27 | 30 | 31 |  | 212 | 12 | 12 | 9 | 12 | 12 | 12 |
| Pilsen, Czech Republic | 174 | 40 | 37 | 25 | 34 | 40 | 42 |  | 165 | 12 | 11 | 9 | 11 | 12 | 12 |
| Padova, Italy | 314 | 40 | 40 | 32 | 39 | 41 | 59 |  | 314 | 12 | 13 | 12 | 13 | 13 | 25 |
| Noordkempen, Belgium | 2580 | 30 | 30 | 19 | 26 | 34 | 40 |  | 1412 | 8 | 8 | 6 | 7 | 9 | 9 |
| Uppsala, Sweden | 1135 | 20–30 | 30 | 20 | 25 | 33 | 38 |  | 1097 | 6–18 | 8 | 6 | 7 | 18 | 21 |
| Copenhagen County, Denmark | 2296 | 40 | 40 | 29 | 39 | 41 | 43 |  | 2142 | 12 | 13 | 11 | 13 | 13 | 13 |
| Dublin, Ireland | 961 | 20 | 19 | 15 | 18 | 20 | 21 |  | 930 | 12 | 12 | 10 | 11 | 12 | 13 |
| Maracaibo, Venezuela | 601 | 40 | 32 | 21 | 28 | 35 | 39 |  | 589 | 12 | 12 | 9 | 11 | 12 | 12 |
| Montevideo, Uruguay | 1772 | 30 | 18 | 11 | 16 | 20 | 21 |  | 1438 | 9 | 6 | 6 | 6 | 7 | 7 |

Daytime was the interval from 10:00 h to 20:00 h in Europeans and South Americans, and from 08:00 h to 18:00 h in Asians. The corresponding nighttime intervals ranged from midnight to 06:00 h and from 22:00 h to 04:00 h, respectively.

**Supplementary Table 5**

Home blood pressure measurement by IDHOCO cohort

| Study cohorts | N° of  people  (n=6887) | Monitoring | | |  | N° of home blood pressure readings | | | | | |
| --- | --- | --- | --- | --- | --- | --- | --- | --- | --- | --- | --- |
|  |  | Device | N° of  days | times  per Day |  | Planned | Median | P5 | P25 | P75 | P95 |
| Ohasama, Iwate, Japan | 2115 | Omron HEM-401C | 28 | 2 (M, E) |  | 56 | 52 | 17 | 40 | 55 | 59 |
| Tsurugaya, Japan | 817 | Omron HEM-722C | 30 | 1 (M) |  | 30 | 13 | 3 | 5 | 26 | 33 |
| Noordkempen, Belgium | 411 | Omron HEM-705CP | 7 | 2 (M, E) |  | 42 | 42 | 24 | 37 | 45 | 54 |
| Didima, Greece | 665 | Omron HEM-705CP | 3 | 2 (M, E) |  | 12 | 12 | 11 | 12 | 12 | 12 |
| Finnish National Sample | 2074 | Omron HEM-722C | 7 | 2 (M, E) |  | 28 | 28 | 16 | 28 | 28 | 28 |
| Buenos Aires, Argentina | 406 | Omron HEM-705CP | 4 | 3 (M, A, E) |  | 24 | 24 | 20 | 24 | 26 | 28 |
| Montevideo, Uruguay | 399 | SpaceLabs 90207 | 1 | 2 (M, E) |  | 2 | 2 | 2 | 2 | 2 | 2 |

Abbreviations: M, morning; A, afternoon; E, evening. All devices passed validation.

**Supplementary Table 6**

Baseline characteristics of IDACO and IDHOCO participants

| Characteristic |  | IDACO |  | IDHOCO |
| --- | --- | --- | --- | --- |
| Number of participants (%) |  |  |  |  |
| All participants in category |  | 12,624 |  | 6887 |
| Ethnicity |  |  |  |  |
| Asian |  | 1883 (14.9) |  | 2932 (42.6) |
| European |  | 8368 (66.3) |  | 3150 (45.7) |
| South American |  | 2373 (18.8) |  | 805 (11.7) |
| Women |  | 6245 (49.5) |  | 3883 (56.4) |
| Smokers |  | 3484 (27.6) |  | 1395 (20.3) |
| Drinking alcohol |  | 5946 (47.1) |  | 2941 (42.7) |
| Obesity |  |  |  |  |
| Body mass index 25.0–29.9 kg/m^2^ |  | 4455 (35.3) |  | 2430 (35.3) |
| Body mass index ≥30.0 kg/m2 |  | 1794 (14.2) |  | 1019 (14.8) |
| On antihypertensive drugs |  | 2315 (18.3) |  | 1803 (26.2) |
| Diabetes mellitus |  | 829 (6.6) |  | 554 (8.0) |
| History of cardiovascular disease |  | 1350 (10.7) |  | 679 (9.9) |
| Mean (±SD) of characteristic |  |  |  |  |
| Age (years) |  | 51.7±16.1 |  | 59.0±14.1 |
| Body mass index (kg/m^2^) |  | 25.5±4.4 |  | 25.7±4.4 |
| Office blood pressure |  |  |  |  |
| Systolic (mm Hg) |  | 131.9±23.1 |  | 134.3±20.2 |
| Diastolic (mm Hg) |  | 79.7±11.9 |  | 79.6±11.6 |
| Ambulatory blood pressure |  |  |  |  |
| 24‑h systolic (mm Hg) |  | 123.9±14.4 |  | … |
| 24‑h diastolic (mm Hg) |  | 74.0±8.7 |  | … |
| Daytime systolic (mm Hg) |  | 129.3±15.1 |  | … |
| Daytime diastolic (mm Hg) |  | 78.8±9.3 |  | … |
| Nighttime systolic (mm Hg) |  | 112.9±15.6 |  | … |
| Nighttime diastolic (mm Hg) |  | 65.1±9.6 |  | … |
| Home blood pressure |  |  |  |  |
| Systolic (mm Hg) |  | … |  | 127.3±18.1 |
| Diastolic (mm Hg) |  | … |  | 76.2±9.9 |
| Biochemical measurements |  |  |  |  |
| Serum cholesterol (mmol/L) |  | 5.56±1.13 |  | 5.41±1.07 |
| Blood glucose (mmol/L) |  | 5.21±1.46 |  | 5.47±1.22 |

Office BP was the average of two consecutive readings. To convert serum cholesterol from mmol per liter to mg per deciliter multiply by 38.3. Diabetes mellitus was a self-reported diagnosis, a fasting or random blood glucose level of ≥7.0 mmol per liter (126 mg per deciliter) or ≥11.1 mmol per liter (200 mg per deciliter) or use of antidiabetic drugs.
